# Role of mitochondria in the regulation of ferroptosis and disease

**DOI:** 10.3389/fmed.2023.1301822

**Published:** 2023-12-13

**Authors:** Cheng Fu, Nan Cao, Sen Zeng, Wenhui Zhu, Xinliang Fu, Wenjun Liu, Shuangqi Fan

**Affiliations:** ^1^College of Animal Science and Technology, Zhongkai University of Agriculture and Engineering, Guangzhou, China; ^2^College of Veterinary Medicine, South China Agricultural University, Guangzhou, China

**Keywords:** ferroptosis, mitochondria, disease, mechanism, drug

## Abstract

Ferroptosis is a distinctive form of iron-dependent cell death characterized by significant ultrastructural changes in mitochondria. Given the crucial involvement of mitochondria in various cellular processes such as reactive oxygen species production, energy metabolism, redox status, and iron metabolism, mounting evidence suggests a vital role of mitochondria in the regulation and execution of ferroptosis. Furthermore, there exists a strong correlation between ferroptosis and various diseases. In this review, we aim to summarize the mechanisms underlying the induction and defense of ferroptosis, emphasizing the influence of mitochondria on this intricate process. Additionally, we provide an overview of the role of ferroptosis in disease, particularly cancer, and elucidate the mechanisms by which drugs targeting mitochondria impact ferroptosis. By presenting a theoretical foundation and reference point, this review aims to contribute to both basic cell biology research and the investigation of clinically relevant diseases.

## 1 Introduction

Mitochondria, commonly known as the powerhouses of the cell, hold a pivotal role in cellular energetics (1). While mitochondria have long been recognized for their crucial role in energy production through oxidative phosphorylation, recent research over the past few decades has unveiled that mitochondria serve a much broader function beyond being mere energy generators (2). These range from maintaining calcium homeostasis and regulating reactive oxygen species (ROS) to orchestrating apoptosis and modulating cellular signaling (3, 4).

Ferroptosis, first proposed by Dr. Brent R. Stockwell of Columbia University in 2012, is a form of programmed non-apoptotic cell death that depends on the presence of iron (5). A distinctive feature of ferroptosis is the significant alteration of mitochondrial structure and function, setting it apart from other forms of cell death (6, 7). When exposed to erastin, the most commonly used reagent for triggering ferroptosis, cells exhibit specific increases in mitochondrial potential and membrane density. This is accompanied by corresponding decreases in volume, the reduction or disappearance of mitochondrial ridges, and the rupture of the mitochondrial outer membrane (8). Notably, ferroptosis lacks the typical morphological features associated with other forms of cell death, such as nuclear concentration, fragmentation, dissolution (cell necrosis), cell shrinkage, chromatin agglutination, apoptotic body formation (apoptosis), or bilayer-membrane encapsulated vesicles (autophagy formation). Mitochondrial damage has been observed in various models, including oxidative glutamate toxicity, glutathione peroxidase consumption, and ferroptosis (9, 10). The utilization of RAS-selective lethal 3 (RSL3) to induce ferroptosis in HT22 cells of neurons and mouse embryonic fibroblasts (MEFs) results in concentration-dependent inhibition of glutathione peroxidase 4 (GPX4), lipid peroxidation, enhanced mitochondrial fragmentation, loss of mitochondrial membrane potential, and decreased mitochondrial respiration (11). Notably, incubating MEFs with RSL3 led to the time-dependent rupture of mitochondrial membranes (12).

## 2 Key mechanisms of ferroptosis: unraveling the pathways and significance

**Figure 1:**
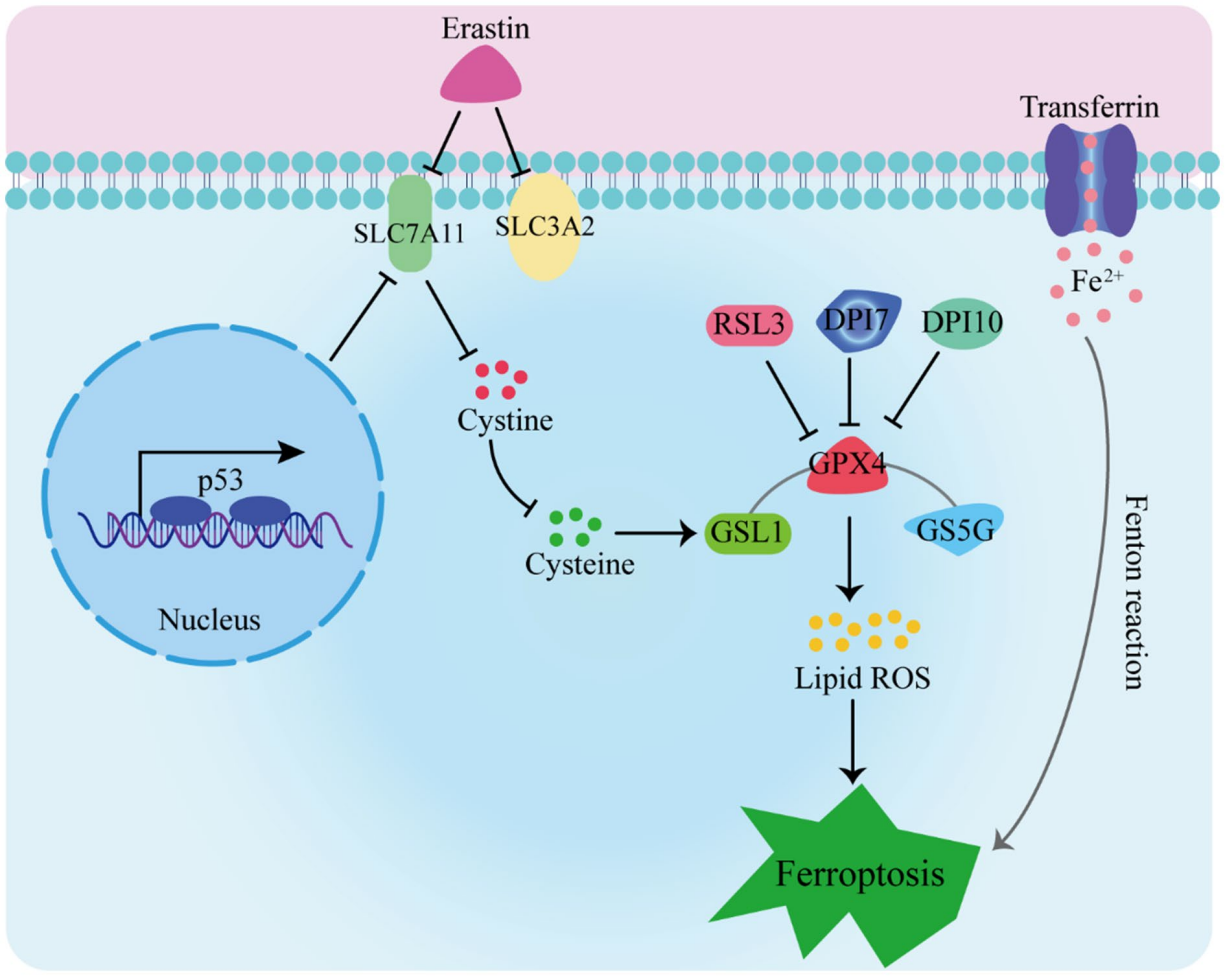
Induction of ferroptosis Pathways.

Ferroptosis has garnered extensive attention due to its distinctive mechanisms. In this manuscript, we explore the primary pathways and their significance in ferroptosis (as depicted in Figure 1):

1. The Glutathione Peroxidase Pathway. The glutathione peroxidase pathway stands as a fundamental element in ferroptosis. GPX4, an inhibitory protein involved in lipid peroxidation, plays a crucial role in preventing ferroptosis. It is responsible for degrading small molecule peroxides and complex lipid peroxides (13–15). Additionally, upstream pathways can impact GPX activity, leading to reduced cellular antioxidant capacity, increased lipid peroxidation, elevated lipid ROS levels, and ultimately culminating in ferroptosis (16–18).
2. The cystine/glutamate transporter pathway also influences ferroptosis (19–21). In organotypic hippocampal culture models (OHSC), glutamate can trigger excitotoxic cell death by modulating calcium influx or inhibiting the cystine absorption pathway (system Xc-) (5). Erastin, on the other hand, inhibits glutathione absorption by targeting system Xc-. Glutathione serves as a vital cofactor for GPXs, thus when its availability is limited, the activity of GPXs is reduced. As a result, the cellular anti-peroxidation ability diminishes, leading to the accumulation of ROS in lipids and subsequent oxidative cell death (22).
3. The Cystine/Glutamate Transporter Pathway. Another key pathway influencing ferroptosis is the cystine/glutamate transporter pathway. Glutamate can trigger excitotoxic cell death in organotypic hippocampal culture models (OHSC) by modulating calcium influx or inhibiting the cystine absorption pathway (system Xc-). Erastin, on the other hand, inhibits glutathione absorption by targeting system Xc-. The limited availability of glutathione, a vital cofactor for GPXs, reduces GPX activity, leading to diminished cellular anti-peroxidation ability, ROS accumulation in lipids, and oxidative cell death (22).
4. The p53 Pathway. The p53 pathway is involved in mediating ferroptosis. The p53 protein inhibits cystine absorption through system Xc- by down-regulating Solute Carrier Family 7 Member 11 (SLC7A11) expression. This reduction in cystine-dependent glutathione peroxidase activity results in increased lipid ROS levels, ultimately leading to ferroptosis in cells (23, 24). Moreover, studies have shown that p53 activation can induce both cell apoptosis and ferroptosis, suggesting its dual role (25).

Furthermore, intracellular iron sources and transport-related proteins, such as hemeoxygenase-1 (HO-1), transferrin, and ferroportin-1 (FPN1), are involved in the regulation of ferroptosis (26, 27). Accumulation of a substantial amount of free Fe^2+^ within cells can induce ferroptosis (28). This is attributed to Fe2+ acting as a cofactor for various metabolic enzymes, thereby enhancing their activity, such as lipid oxygenase, and promoting the generation of lipid peroxides. Additionally, the Fenton reaction, catalyzed by Fe2+, leads to the production of peroxyradicals and hydroxyl radicals, which further react with lipid peroxides, resulting in the generation of a significant number of lipid ROS. Ultimately, these processes culminate in the induction of cell ferroptosis.

5. Intracellular Iron Regulation. Intracellular iron sources and transport-related proteins, such as hemeoxygenase-1 (HO-1), transferrin, and ferroportin-1 (FPN1), are integral to the regulation of ferroptosis (26, 27). Accumulation of free Fe^2+^ within cells can induce ferroptosis due to its role as a cofactor for various metabolic enzymes, enhancing their activity, including lipid oxygenase. The Fenton reaction, catalyzed by Fe^2+^, leads to the production of peroxyradicals and hydroxyl radicals, which react with lipid peroxides, generating a significant amount of lipid ROS and culminating in cell ferroptosis (28).

RSL3, DPI7, and DPI10 directly impact GPXs’ activity, leading to a reduction in cellular antioxidant capacity, an increase in lipid peroxidation, elevated levels of lipid reactive oxygen species (L-ROS), and ultimately culminating in ferroptosis. Erastin inhibits cysteine entry into cells by targeting system Xc-, resulting in a decrease in intracellular glutathione (GSH) levels. This decrease leads to reduced GPX4 activity, disruption of cellular redox homeostasis, and the accumulation of L-ROS, ultimately causing iron-dependent cell death. Additionally, transferrin binds to the transferrin receptor, facilitating the transport of free iron to various tissues and cells. This process contributes to iron-dependent cell death through the Fenton reaction.

## 3 The role of mitochondria-related iron metabolism, ROS generation, and hydrogen peroxide induction in ferroptosis

**Figure 2:**
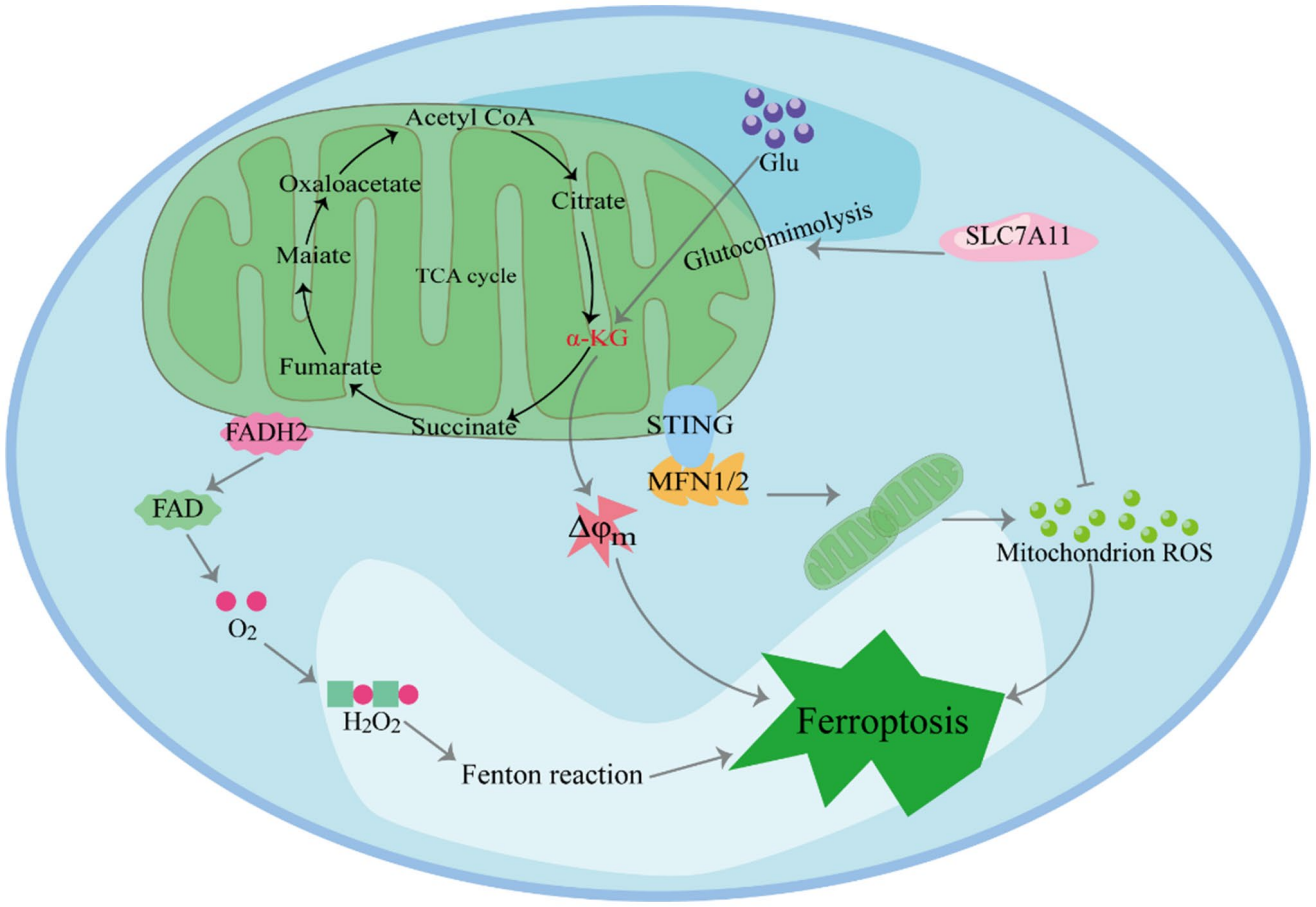
The role of mitochondria in inducing ferroptosis.

Mitochondria, central to iron metabolism, play a crucial role in the induction of ferroptosis (Figure 2) (29–31). In human pancreatic cancer cells, it has been observed that the endoplasmic reticulum protein stimulator of interferon genes (STING1) accumulates in mitochondria and interacts with the mitochondrial outer membrane protein mitofusins (MFN1/2). This interaction triggers mitochondrial fusion, leading to the subsequent production of ROS and lipid peroxidation, ultimately promoting ferroptosis. Furthermore, *in vitro* studies and xenografted mouse models have shown that the deletion of the STING1 or MFN1/2 genes reduces the sensitivity of pancreatic cancer cells to ferroptosis (32).Cellular ROS primarily originate from mitochondrial metabolism and serve as mediators in intracellular signal transduction. The key ROS species include hydrogen peroxide (H2O2), superoxide anion, hydroxyl radical and peroxynitrite (33). The accumulation-derived ROS renders cells susceptible to ferroptosis (34, 35). For example, in lipopolysaccharide-induced acute kidney injury (AKI), mitochondria-derived ROS contribute to ferroptosis in kidney cells (36). Research has shown that ROS generated by mitochondrial respiration can damage enzymes in the mitochondrial respiratory chain complex. Firstly, inhibition of SLC7A11 or deprivation of cystine significantly reduces in GSH, weakening its ability to clear ROS (37). Secondly, SLC7A11 inactivation leads to the accumulation of glutamate in cells, which is converted to α-ketoglutaric acid (α-KG). This conversion promotes ferroptosis by enhancing tricarboxylic acid (TCA) cycling, ultimately resulting in hyperpolarization of the mitochondrial membrane potential (38–40).

The leakage of electrons from electron transport chain (ETC) complexes I and III gives rise to superoxide anions, which are subsequently converted to H2O2 through superoxide dismutase (SOD)-mediated disproportionation. The reaction between H2O2 and unsaturated fatty acids containing Fe^2+^ results in the formation of polyunsaturated fatty acid free radicals (41). These unstable carbon core free radicals rapidly react with oxygen to generate polyunsaturated fatty acids (PUFAs) peroxygen free radicals, consequently triggering cell death.

In the investigation of cysteine deprivation-induced (CDI) ferroptosis, the inhibition of mitochondrial complex III function using electron leakage site IIIQo inhibitors has been shown to reduce lipid peroxidation and ferroptosis in mouse hepatocellular carcinoma cells (42). Furthermore, various ETC inhibitors and mitochondrial uncoupling agents have demonstrated an inhibitory effect on ferroptosis in CDI (31). Moreover, mitochondria play a central role in cellular metabolism, and the breakdown of mitochondrial glutamine can promote ferroptosis under conditions of amino acid starvation. Studies have emphasized the pivotal role of mitochondria in cysteine-deprivation-induced ferroptosis, distinct from the GPX4-mediated ferroptosis pathway. Mechanistically, glutamine contributes to iron-dependent cell death through mitochondrial TCA cycling and the ETC. Blocking these processes effectively inhibits the accumulation of lipid peroxides and iron-dependent cell death (43).

Changes in mitochondrial TCA cycling and glycolysis play a pivotal role in promoting ferroptosis. Studies have revealed that increasing TCA cycling activity heightens cell sensitivity to CDI ferroptosis (34, 43). Moreover, key enzymes within the TCA cycle regulate ferroptosis. For instance, the α-ketoglutarate (α-KG) dehydrogenase complex (KGDHC) catalyzes the oxidative decarboxylation of α-KG to succinyl-CoA during the TCA cycle, and its inactivation inhibits the occurrence of ferroptosis. However, supplementation of α-ketoglutarate and downstream metabolites of 2-oxoglutarate dehydrogenase complex (OGDC) mimics the function of glutamine in CDI ferroptosis. Furthermore, mitochondria-mediated glutamine decomposition is involved in various reactions that support the TCA cycle, and reducing or inhibiting glutamine decomposition significantly inhibits ferroptosis in CDI (44). In the absence of glutamine, cysteine starvation, or treatment with erastin, mitochondrial damage and cell death cannot be induced.

Glycolysis is one of the primary ways in which cells produce energy without oxygen. However, in tumor cells, glycolysis is favored even in aerobic environments (45). During erastin-induced ferroptosis, a decrease in glycolytic flux was observed in cells with disrupted glycolysis (46), leading to enhanced oxidative phosphorylation from mitochondria, ultimately resulting in ferroptosis (47). Similarly, studies on ferroptosis induced by RSL3 detected dysfunctional glycolytic function and down-regulated levels of three key glycolytic enzymes: hexokinase II, platelet-type phosphofructose kinase, and pyruvate kinase M2 (48). Additionally, literature has reported the involvement of various mitochondrial respiratory enzymes, such as citrate synthetase (CS), cytochrome c oxidase 2, and fumarate hydratase (FH), in the regulation of ferroptosis (43). Notably, FH has been identified as a tumor suppressor that increases cell sensitivity to CDI ferroptosis. The alteration of voltage-dependent anion-selective channel (VDAC) function may be one of the reasons for changes in glycolysis. VDAC is located in the outer membrane of mitochondria and plays a crucial role in regulating mitochondrial metabolism, productivity, and cell survival and death signaling. It acts as a transmembrane channel for transporting ions and metabolites, including its subtypes VDAC1, VDAC2 and VDAC3. When VDAC is open, it allows respiratory substrates, adenosine diphosphate (ADP), and phosphoric acid to enter mitochondria. Conversely, when it is closed, it blocks mitochondrial transport function (49). The dynamic “open-closed” state significantly impacts mitochondrial metabolism and cellular bioenergy. Erastin can attenuate the influence of free tubulin on VDACs, leading to an increase in mitochondrial membrane potential through the opening of VDACs. Consequently, this elevated potential contributes to the augmented formation of ROS (50). Cells with a higher abundance of VDACs exhibit increased sensitivity to erastin. Furthermore, the opening of VDACs and the subsequent overproduction of ROS result in a mitochondrial calcium ion overload (51). This overload promotes the opening of mitochondrial permeability transition pores, exacerbating the decline of mitochondrial transmembrane potential and leading to subsequent ATP depletion.

Mitochondria play a crucial role in H2O2-induced ferroptosis (52). The enzymes involved in ferroptosis include protochlorophyllide oxidoreductase and cytochrome b5 reductase 1 (CYB5R1) (53). These enzymes transfer electrons from nicotinamide adenine dinucleotide phosphate (NAD(P)H) to downstream proteins, such as cytochrome P450 (CYP450). During this electron transfer process, a small fraction of electrons “accidentally” gets transferred to oxygen present in the solution. Oxygen accepts these electrons and is converted to H2O2. In the presence of iron ions, H2O2 undergoes the Fenton reaction, generating hydroxyl free radicals. These active hydroxyl radicals target the hydrogen atoms between the long-chain double bonds of unsaturated fatty acids, leading to lipid peroxidation (54, 55). In experiments using cells lacking mitochondrial DNA, researchers discovered that these cells exhibit high levels of Fe^2+^, resulting in the production of hydroxyl radicals from hydrogen peroxide. Aquaporins (AQP) 3, 5, and 8 bind to nicotinamide adenine dinucleotide phosphate oxidase 2 (NOX2) and regulate the permeability of extracellular hydrogen peroxide. This regulation ultimately contributes to the occurrence of ferroptosis (56).

## 4 Mitophagy, O-GlcNAcylation and mitochondrial DNA stress involvement in ferroptosis

Mitophagy, the selective degradation of mitochondria through autophagy, plays a pivotal role in ferroptosis. O-GlcNAcylation modification, acting as a nutrient and stress receptor in cells, rapidly increase under the stress conditions of ferroptosis. Inhibition of O-GlcNAcylation can promote iron autophagy, leading to the release of iron stored in ferritin. The released iron is then transported to mitochondria as a buffer, promoting mitophagy, subsequent iron release from mitochondria, and ultimately facilitating the process of ferroptosis (57). Consequently, the high concentration of iron makes mitochondria ideal sites for inducing ferroptosis. Experiments on HEK293T cells have demonstrated that mitochondrial oxidative stress mediates iron-induced ferroptosis through the NF-E2-related factor 2 (Nrf2)-antioxidant response element (ARE) pathway (58). Moreover, studies have shown that atorvastatin, a medication used to lower cholesterol, induces mitochondria-dependent ferroptosis by modulating the Nrf2-xCT/GPx4 axis (59). In addition, mitochondrial DNA stress activates the STING1/ transmembrane protein173 (TMEM173)-mediated DNA sensing pathways, leading to autophagy-dependent ferroptosis through lipid peroxidation (60). Furthermore, the loss of mitochondrial DNA and subsequent mitochondrial dysfunction can increase the sensitivity of liver cells to ferroptosis under conditions of iron overload. Additionally, mitochondrial DNA stress triggers autophagy-dependent ferroptosis by activating the cyclic GMP-AMP synthase (cGAS)-STING1 pathway (61, 62).

Mitochondria play a crucial role in inducing ferroptosis. First, SLC7A11 inactivation leads to the accumulation of glutamate in cells, which is converted to α-KG. This conversion promotes ferroptosis by enhancing TCA cycling, ultimately resulting in hyperpolarization of the mitochondrial membrane potential. Furthermore, Mitochondria play a crucial role in H2O2-induced ferroptosis. In the presence of iron ions, H2O2 undergoes the Fenton reaction, generating hydroxyl free radicals. These active hydroxyl radicals target the hydrogen atoms between the long-chain double bonds of unsaturated fatty acids, leading to lipid peroxidation. Moreover, STING1 accumulates in mitochondria and interacts with MFN1/2. This interaction triggers mitochondrial fusion, leading to the subsequent production of ROS and lipid peroxidation, ultimately promoting ferroptosis.

## 5 Cellular defense mechanisms against ferroptosis

**Figure 3:**
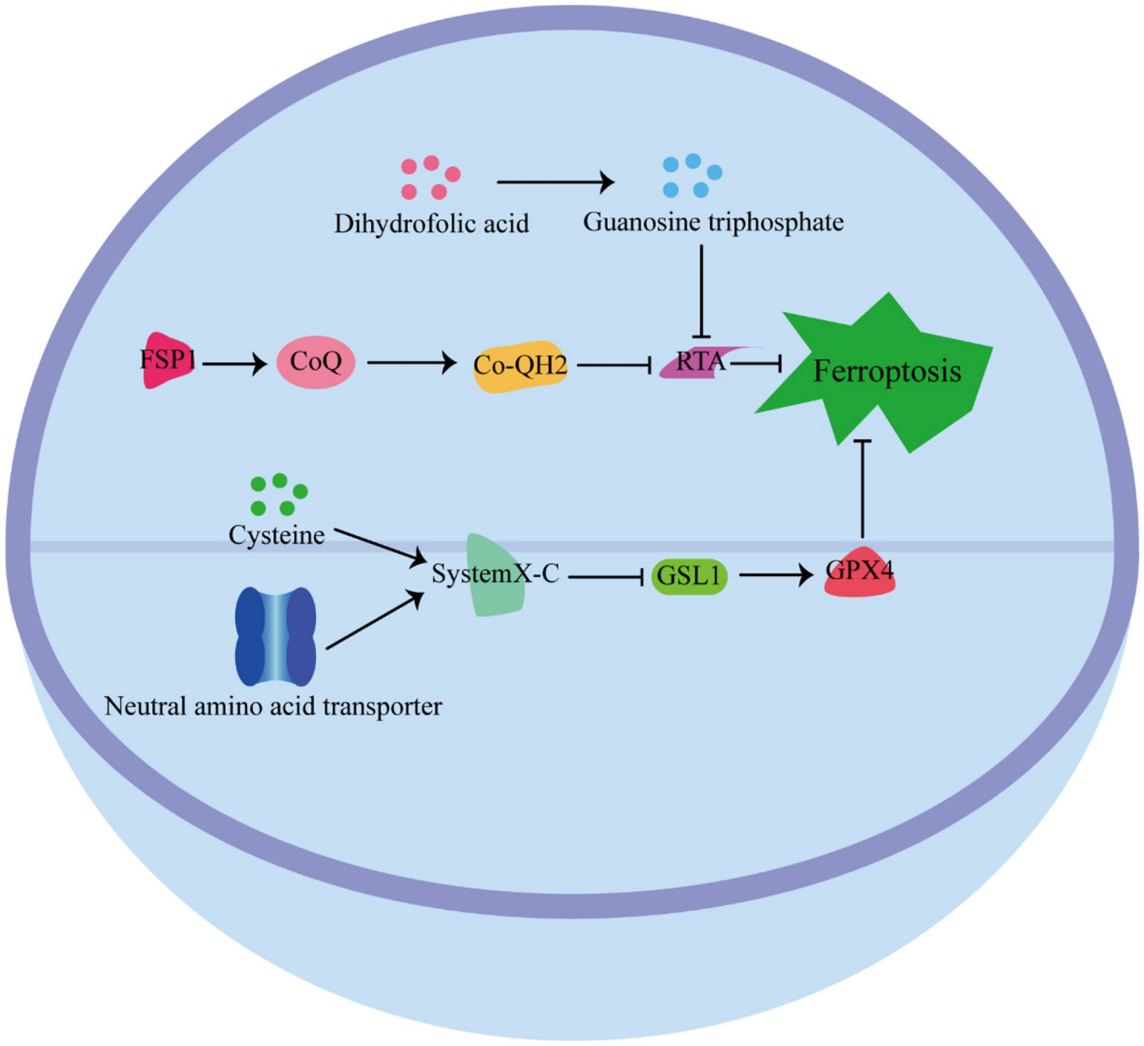
Pathways defense ferroptosis.

Cells have developed various defense mechanisms to counteract ferroptosis (Figure 3) (63). One of the most well-known defense pathways against ferroptosis is mediated by GPX4. GPX4 specifically catalyzes the reduction of lipid peroxides in a GSH-dependent manner, thereby preventing their accumulation and protecting cells from ferroptosis (64). The niacinamide adenine dinucleotide (NAD)/ferroptosis suppressor protein 1 (FSP1)/Coenzyme Q_10_ (CoQ_10_) system is the second defense pathway against ferroptosis (65). FSP1 is catalyzed by NAD(P)H, and inhibits ferroptosis vis ubiquinone CoQ_10_. CoQ_10_ acts as a potent antioxidant capable of capturing lipid peroxidation free radicals, thereby inhibiting ferroptosis (66). In addition, the tetrahydrobiopterin (BH4)/dihydrofolate reductase (DHFR) system is another defense mechanism against lipid peroxidation and ferroptosis. Guanosine triphosphate cyclohydrolase 1 (GCH1), the rate-limiting enzyme in tetrahydrobiopterin biosynthesis, has been identified as a ferroptosis inhibitor independent of GPX4 (67). The BH4/DHFR system relies on the regeneration of BH4 through the action of DHFR. Unlike FSP1 or GCH1 inhibition alone, which is not sufficient to induce ferroptosis, their inhibition significantly increases the sensitivity of cells to.

The pathway by which cells resist iron death. GPX4 Pathway: GPX4 relies on GSH to catalyze the reduction of lipid peroxides to protect cells from ferroptosis. NAD/FSP1/CoQ10 pathway: FSP1 is catalyzed by NAD, and inhibits ferroptosis vis ubiquinone CoQ10, which acts as a potent antioxidant capable of capturing lipid peroxidation free radicals to inhibit ferroptosis. BH4/ DHFR pathway: BH4 through the action of DHFR against lipid peroxidation and ferroptosis.

## 6 The role of mitochondrial integrity, metabolism, and activity against ferroptosis

**Figure 4:**
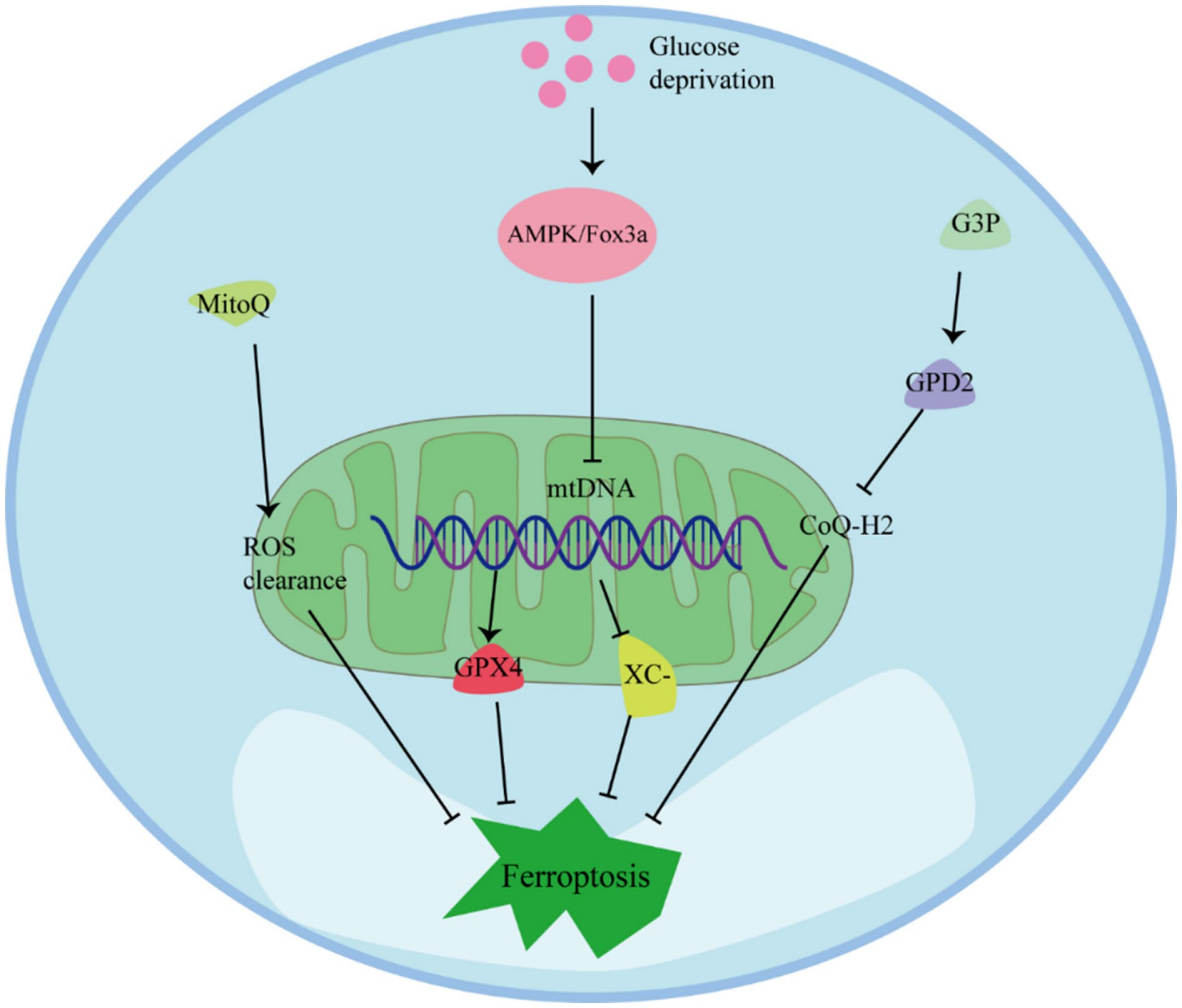
The role of mitochondria against ferroptosis.

Mitochondria play multiple regulatory roles in ferroptosis and are crucial for cellular resistance against it (Figure 4) (68). Preserving mitochondrial integrity is considered an effective strategy to prevent ferroptosis in various cell types. Inhibition of BH3-interacting domain death agonist (BID) or maintaining mitochondrial integrity and function through mitochondria-targeting ROS clearance agents like MitoQ has been shown to effectively prevent ferroptosis in different cell types (44). In cells with depleted mitochondrial DNA (mtDNA), the expression of antioxidant enzymes and mitochondrial GPX4 is upregulated, and the system Xc^−^ channel is inhibited by erythropoietin, resulting in enhanced resistance to ferroptosis (69). Additionally, under conditions of GPX4 inactivation, the mitochondrial enzyme dihydroorotate dehydrogenase (DHODH) can protect cells from ferroptosis by inhibiting lipid peroxidation (70). Therefore, maintaining fully functioning mitochondria is crucial for cellular resistance against ferroptosis (71).

Studies have shown that glucose deprivation also triggers the induction of forkhead box O3 (FoxO3a), resulting in a decrease in mitochondrial-associated genes, mtDNA copy number, mitochondrial protein expression, and levels of respiratory complexes. The absence of FoxO3a promotes mitochondrial membrane potential hyperpolarization, increased oxygen consumption, accumulation of lipid peroxidation, and eliminates the protective effect of energy stress on ferroptosis *in vitro*. These findings suggest that energy stress, mediated through the AMP-activated protein kinase (AMPK)/FoxO3a signaling pathway, modulates mitochondrial activity and influences the occurrence of ferroptosis (72, 73).

Regulation of mitochondrial metabolism and activity can play a role in inhibiting ferroptosis. Fatty acid β-oxidation, which primarily occurs in mitochondria, can inhibit lipid peroxidation by reducing the accumulation of PUFAs (74). Moreover, glucose deprivation increases in the intracellular AMP/ATP ratio, activating AMPK, which can inhibit ferroptosis induced by compounds such as erastin or RSL3 (75). AMPK has been identified as closely related to the ETC in inhibiting the occurrence of ferroptosis. Mitochondria transfer electrons through the ETC complex, generating a proton concentration gradient, which is utilized by ATP synthase to produce energy in the form of ATP. Increased ATP production inhibits AMPK activity, thereby promoting ferroptosis through reduced phosphorylation of acetyl-CoA carboxylase (ACC) and increased lipid synthesis. Inactivating ACC prevents ferroptosis (72).

## 7 Mitochondrial regulation of CoQ and protective enzymes against ferroptosis

Mitochondria serve a protective function against ferroptosis by regulating the synthesis and transport of CoQ. The lipid transporter StAR-related lipid transfer domain protein 7 (STARD7) is essential for both CoQ synthesis in mitochondria and its transport to the cell membrane (76). STARD7 in mitochondria plays a protective role in maintaining oxidative phosphorylation and mitochondrial ridge morphogenesis. Meanwhile, STARD7 in the cytoplasm is responsible for transporting CoQ to the cell membrane and preventing iron oxidation. Interestingly, overexpression of STARD7 in the cytoplasm can increase cellular resistance to ferroptosis (77). Another enzyme, Glycerol-3-phosphate dehydrogenase 2 (GPD2), also contributes to the protection against ferroptosis by inhibiting mitochondrial lipid peroxidation. GPD2 produces reduced CoQ-H2 in the inner membrane of mitochondria, thereby defending against cellular ferroptosis. Metabolome analysis has shown that treatment with GPX4 inhibitors leads to a loss of glycerol-3-phosphate (G3P) in cells. Further investigation revealed that cancer cells supplemented with G3P attenuated ferroptosis induced by GPX4 inhibitors in a GPD2-dependent manner. Deletion of GPD2 in cancer cells increased their sensitivity to mitochondrial lipid peroxidation and ferroptosis caused by GPX4 inhibition. Moreover, simultaneous knockdown of GPX4 and GPD2 synergistically inhibited tumor growth driven by ferroptosis (78).

Many drugs and molecules have been identified to regulate ferroptosis through their interactions with mitochondria (Table 1). For example, Cannabidiol has been found to inhibit oxytosis/ferroptosis by directly targeting mitochondria, independent of cannabinoid receptors (79). N-acetylcysteine has been demonstrated to alleviate ferroptosis in diabetic nephropathy by maintaining mitochondrial redox homeostasis, It achieves this by activating the Sirtuin 3 (SIRT3)- SOD2/GPX4 pathway (80). Besides, in AKI, HIF may protect kidney cells in AKI from ferroptosis by reducing mitochondrial oxidative stress and damage (81).

**Table 1:** Major drugs/targets and their effects on ferroptosis aimed at mitochondria.

Drugs/reagents targeting mitochondria	Effect on ferroptosis	Role in diseases
STING1	Promotion	/
MFN1/2	Promotion	/
ROS	Promotion	/
Glutamine	Promotion	/
Glutamate	Promotion	/
α-KG	Promotion	/
PUFA hydroperoxide	Promotion	/
Cys	Promotion	/
Erastin	Promotion	/
KGDHC	Promotion	/
VDAC	Promotion	/
SLC7A11	Promotion	/
Arsenic exposure	Promotion	Inducing acute lung injury
FUNDC1	Promotion	Inducing liver injury
SSBP1	Promotion	Inhibiting progression and migration of GBM cells
AC	Promotion	Exhibiting anti-tumor activity
COX7A1	Promotion	/
Electron leakage site IIIQo inhibitors	Inhibition	/
ETC inhibitors	Inhibition	/
Mitochondrial uncoupling agents	Inhibition	/
MitoQ	Inhibition	/
Erythropoietin	Inhibition	/
DHODH	Inhibition	/
FoxO3a	Inhibition	/
AMPK	Inhibition	/
ACC	Inhibition	/
STARD7	Inhibition	/
CoQ	Inhibition	/
GPD2	Inhibition	/
Cannabidiol	Inhibition	/
N-acetylcysteine	Inhibition	/
NOX4	Inhibition	Delaying Alzheimer’s disease
Ferrostatin-1	Inhibition	Improving IIRI and UC
Desferrioxamine	Inhibition	Improving UC

Mitochondria play a protective role against ferroptosis through several mechanisms. MitoQ and ROS Regulation: MitoQ is a mitochondria-targeted antioxidant that can help prevent ferroptosis by reducing mitochondrial-derived ROS. AMPK Activation: Glucose deprivation can lead to an increase in the intracellular AMP/ATP ratio, which activates the AMPK pathway. AMPK can inhibit ferroptosis induced by compounds like erastin or RSL3. FoxO3a and mtDNA Regulation: Under conditions of glucose deprivation, the induction of FoxO3a results in a decrease in mtDNA. Depletion of mtDNA can trigger various cellular responses, including the upregulation of antioxidant enzymes and mitochondrial GPX4, which are key players in protecting cells from ferroptosis. G3P-GPD2 pathway: GPD2, which operates within the inner mitochondrial membrane, plays a protective role by inhibiting mitochondrial lipid peroxidation. It produces reduced CoQ-H2 in mitochondria, thereby defending against ferroptosis.

## 8 Dual role of ferroptosis in disease pathology and therapeutic potential

Ferroptosis occurs in myofibroblasts during fibroblast activation and extracellular matrix deposition in the fibrotic cascade. Pharmacological modulation of ferroptosis has shown promising therapeutic effects on renal fibrosis (82, 83). Ferroptosis plays a crucial role in the occurrence and development of intestinal diseases, particularly in intestinal ischemia–reperfusion injury (IIRI), inflammatory bowel disease (IBD), and colorectal cancer. In the context of IIRI, intestinal epithelial cells of mice experience a decrease in GSH content and GSH/GSSG ratio, alongside a reduction in mRNA and protein expression levels of GPX4 and FTH1, which are key regulators of ferroptosis. Concurrently, protein expression levels of cyclooxygenase-2, a marker of ferroptosis, and concentrations of malondialdehyde and Fe^2+^ increase. Treating mice with ferrostatin-1, a ferroptosis inhibitor, improves IIRI, suggesting that targeting iron death could be a promising strategy for treating IIRI (84). Similarly, in experimental animal models and patients with IBD, intestinal epithelial cells also exhibit characteristics of iron death. Researchers have observed significant changes in genes related to ferroptosis in the intestinal epithelial cells of ulcerative colitis (UC) patients and UC model mice. There is also a notable increase in malondialdehyde, ROS, and iron levels, along with up-regulated expression of ferritin heavy chain/light-chain (FTH1/FTL). Symptoms in UC mice were reduced after treated with iron death inhibitors such as Ferrostatin-1 and desferrioxamine. These findings indicate the pivotal involvement of ferroptosis in IBD, suggesting that inhibiting iron death could be a potential therapeutic approach (85).

Ferroptosis has been shown to inhibit tumor growth and increase the sensitivity of many tumors to chemotherapy and immunotherapy (86, 87). Loss of tumor suppressor factors such as p53 and BRCA1 associated protein 1 (BAP1), mutations in proto-oncogenes like KRAS, or overexpression of pro-tumor functional proteins such as OTU domain, ubiquitin aldehyde binding 1 (OTUB1) can lead to an increase in the level of SLC7A11, which is the cystine transporter also known as xCT (19). Because of its involvement in tumor growth and response to therapy, ferroptosis can be targeted for drug action in cancer treatment (88, 89). Modulating the pathways and factors involved in ferroptosis, such as targeting GPX4, can induce ferroptosis in cancer cells and offer potential therapeutic opportunities (90). Indeed, classical tumor suppressor p53 has been shown to mediate the process of ferroptosis and inhibit tumor growth. Ferroptosis has been suggested to act as an adaptive response to metabolic imbalances, offering a promising approach for targeting and eradicating malignant cells (91, 92). Researchers have utilized the ubiquitination of GPX4 to identify a small molecule that serves as an inducer of ferroptosis and apoptosis in triple-negative breast cancer cells (93).

## 9 Ferroptosis in disease therapy through targeting mitochondrial

In cardiovascular and cerebrovascular diseases, as well as neurodegenerative diseases, ferroptosis may cause damage to normal tissues and organs through mitochondria. Inhibiting ferroptosis has been shown to delay the onset of these diseases (94). For instance, in Alzheimer’s disease, NADPH Oxidase 4 (NOX4) promotes ferroptosis in astrocytes by inducing oxidative stress-induced lipid peroxidation and impairing mitochondrial metabolism (95). Arsenic exposure induces ferroptosis and acute lung injury through the production of mitochondrial reactive oxygen species (MTROS) and subsequent mitochondrial-associated endoplasmic reticulum dysfunction (20). Ferroptosis also plays a significant role in liver and kidney pathology. Studies have revealed that the mitochondrial phagocytic receptor FUN14 domain containing 1 (FUNDC1) directly interacts with GPX4 to control liver iron necrosis and fibrosis injury through mitochondrial phagocytic dependence. FUNDC1 is involved in liver injury induced by carbon tetrachloride (CCL4) exposure (96). Recent research has demonstrated that targeting ferroptosis can effectively alleviate chronic kidney injury and renal fibrosis.

Using a novel mitochondrial-associated gene risk model and *in vitro* experiments, researchers have found that knockdown of the Single-stranded DNA-binding protein 1 (SSBP1) gene leads to mitochondrial dysfunction and increased levels of ROS. This, in turn, inhibits the progression and migration of glioblastoma (GBM) cells by enhancing ferroptosis. SSBP1 has been identified as a potential ferroptosis-associated biomarker for GBM (97).Ferroptosis regulated by mitochondria plays a crucial role in cancer progression (98, 99). Recent studies have revealed that cGAS is localized to the outer mitochondrial membrane, where it interacts with dynein-associated protein 1 (DRP1) to promote DRP1 oligomerization. This interaction leads to mitochondrial ROS accumulation and an increase in ferroptosis, resulting in the inhibition of tumor growth in the absence of cGAS or DRP1 oligomerization (100). Several anti-tumor drugs have been found to act through the mitochondria-mediated ferroptosis pathway. For example, Avitinib (AC), a novel epidermal growth factor receptor tyrosine kinase inhibitor, exhibits potent anti-tumor activity. AC induces ferroptosis in MCF-7 cells by elevating ferrous ions and ROS levels. Notably,mitochondrial depletion significantly inhibits AC-induced cytotoxicity, including ferroptosis and apoptosis, underscoring the pivotal role of mitochondria in AC-induced ferroptosis and apoptosis (101). Furthermore, cytochrome c oxidase polypeptide 7A1 (COX7A1), a subunit of cytochrome c oxidase, has been implicated in cancer metabolism and therapy. Studies have shown that COX7A1 enhances the sensitivity of non-small cell lung cancer (NSCLC) cells to cysteine-deprivation-induced ferroptosis by promoting TCA cycle activity and the function of mitochondrial complex IV in the ETC (102).

## 10 In summary

Mitochondria play diverse roles in the regulation of cell ferroptosis, and their involvement is closely linked to various diseases and targeted therapies. Understanding the novel functions and mechanisms of mitochondria in ferroptosis provides a valuable theoretical foundation and reference for basic research in cell biology. By elucidating the intricate interplay between mitochondria and ferroptosis, researchers can uncover new insights into disease pathogenesis and identify potential therapeutic targets. This knowledge can pave the way for the development of innovative strategies to modulate ferroptosis and mitigate its impact on human health.

## Author contributions

CF: Conceptualization, Writing – original draft. NC: Writing-review & editing. SZ: Software, Writing – review & editing. WZ: Formal analysis, Writing – review & editing. XF: Formal analysis. WL: Data curation. SF: Conceptualization, Supervision, Writing – original draft. NC: Writing – review & editing. XF: Formal analysis. WL: Data curation.
